# Impact of multiple balloon inflations during primary percutaneous coronary intervention on infarct size and long-term clinical outcomes in ST-segment elevation myocardial infarction: real-world postconditioning

**DOI:** 10.1007/s00395-014-0403-3

**Published:** 2014-01-31

**Authors:** Tuncay Yetgin, Michael Magro, Olivier C. Manintveld, Sjoerd T. Nauta, Jin M. Cheng, Corstiaan A. den Uil, Cihan Simsek, Ferry Hersbach, Ron T. van Domburg, Eric Boersma, Patrick W. Serruys, Dirk J. Duncker, Robert-Jan M. van Geuns, Felix Zijlstra

**Affiliations:** 1 Department of Cardiology, Thoraxcentre, room Ee-2389a, Erasmus University Medical Center, Dr. Molewaterplein 50-60, 3015 GE Rotterdam, The Netherlands; 2 Interuniversity Cardiology Institute of the Netherlands, ICIN-KNAW, Utrecht, The Netherlands; 3 Department of Cardiology, Maasstad Ziekenhuis, Rotterdam, The Netherlands

**Keywords:** Postconditioning, Primary percutaneous coronary intervention, Reperfusion injury, ST-segment elevation myocardial infarction

## Abstract

Interrupting myocardial reperfusion with intermittent episodes of ischemia (i.e., postconditioning) during primary percutaneous coronary intervention (PPCI) has been suggested to protect myocardium in ST-segment elevation myocardial infarction (STEMI). Nevertheless, trials provide inconsistent results and any advantage in long-term outcomes remains elusive. Using a retrospective study design, we evaluated the impact of balloon inflations during PPCI on enzymatic infarct size (IS) and long-term outcomes. We included 634 first-time STEMI patients undergoing PPCI with an occluded infarct-related artery and adequate reperfusion thereafter and divided these into: patients receiving 1–3 inflations in the infarct-related artery [considered minimum for patency/stent placement (controls); *n* = 398] versus ≥4 [average cycles in clinical protocols (postconditioning analogue); *n* = 236]. IS, assessed by peak creatine kinase, was lower in the postconditioning analogue group compared with controls [median (interquartile range) 1,287 (770–2,498) vs. 1,626 (811–3,057) UI/L; *p* = 0.02], corresponding to a 21 % IS reduction. This effect may be more pronounced in women, patients without diabetes/hypercholesterolemia, patients presenting within 3–6 h or with first balloon re-occlusion ≤1 min. No differences were observed in 4-year mortality or MACCE between groups. Four or more inflations during PPCI reduced enzymatic IS in STEMI patients under well-defined conditions, but did not translate into improved long-term outcomes in the present study. Large-scale randomized trials following strict postconditioning protocols are needed to clarify this effect.

## Introduction

In patients presenting with ST-segment elevation myocardial infarction (STEMI), infarct size (IS) can be limited by early myocardial reperfusion via primary percutaneous coronary intervention (PPCI), thereby preserving left ventricular systolic function and improving clinical outcome. However, the full benefits of myocardial reperfusion are not realized, given that the process of restoring blood flow to the ischemic myocardium can independently induce cell death (i.e., lethal reperfusion injury) [13]. Hence, cardioprotective strategies that potentially modify the conditions of reperfusion and limit reperfusion injury are of major clinical interest.

Ischemic postconditioning (IPOC) is an interventional strategy in which controlled, brief, intermittent episodes of re-occlusions in the first few minutes of reperfusion can protect myocardium from lethal reperfusion injury [28]. The concept of interrupting the myocardial reperfusion process with the angioplasty balloon to reduce myocardial IS (as assessed by different modalities, including cardiac biomarkers) has been demonstrated to be efficacious in several small-size studies evaluating STEMI patients undergoing PPCI [10, 19, 21, 26]. However, recent trials provide inconsistent results [4–6, 18, 20]. The efficacy of IPOC appears to be hampered by various confounders and additionally, uncertainties remain about the optimal protective IPOC protocol [12]. More importantly, the effect of IPOC on long-term clinical endpoints such as mortality or major adverse cardiac and cerebrovascular events (MACCE) remains unknown.

In the absence of adequately powered clinical trials, we aimed to investigate the impact of multiple balloon inflations during PPCI on enzymatic IS in patients with STEMI. Accordingly, we aimed to determine whether this may have served as a real-world analogue for IPOC. Secondly, to assess potential confounders, we evaluated whether these effects differed in prespecified subgroups. Finally, we examined the impact of balloon inflations during PPCI on long-term clinical outcome.

## Methods

### Study design and population

As the principal regional cardiac referral center, an ongoing registry of catheter-based coronary procedures is maintained in an electronic database at our institution. We conducted a retrospective chart review, together with the electronic medical records, of all patients who presented to our institution between June 2006 and June 2010 with STEMI undergoing PPCI. We included patients if they: were ≥18 years of age; had symptoms suggesting acute myocardial ischemia lasting >30 min and ST-segment elevation >0.1 mV in ≥2 contiguous leads; had an occluded infarct-related artery (IRA) with TIMI-0/1 flow on initial angiogram; had adequate reperfusion after PCI (TIMI-3). We excluded patients with previous myocardial infarction or CABG, cardiogenic shock, cardiac arrest or thrombectomy. The current retrospective study design was modeled after the study by Darling and colleagues [3].

### Ethics

Patients were not subject to acts for the purpose of this retrospective study. Neither was any mode of behavior imposed, otherwise than their regular treatment. Therefore, according to Dutch law, written informed consent for a patient to be enrolled in this study was not required. This study was conducted according to the Erasmus MC Privacy Policy and regulations for the appropriate use of data in patient oriented research and was approved by the institutional ethics committee.

### Procedures and medications

All procedures were performed following standard procedural guidelines at the time. Intraprocedural anticoagulation was ensured using unfractionated heparin (to achieve an activated clotting time >250 s in all patients). All patients received an aspirin loading dose of 300 mg and were encouraged to continue this regimen indefinitely. After a 600 mg clopidogrel loading dose, additional antiplatelet therapy with a 75 mg clopidogrel maintenance dose was instituted in all patients, who were then advised to continue this regimen for 12 months. In all cases, the interventional strategy, including pre-dilatation, post-dilatation, glycoprotein IIb/IIIa inhibitors (to prevent or to treat distal microembolization) and other medications such as adenosine, was at the discretion of the interventional cardiologist.

### Balloon inflations

In the currently available clinical trials, the IPOC protocols consisted of 2–4 cycles of ischemia and reperfusion (produced by inflations/deflations of angioplasty balloon) after direct stenting [27]. Taking into account an average of three cycles utilized in the clinical protocols plus one balloon inflation for direct stenting, we reasoned that ≥4 inflations would mimic an IPOC stimulus in a real-world setting. Thus, the study population was divided into patients receiving 1–3 balloon inflations during PPCI in the IRA (considered minimum range for achieving patency/stent placement [controls]) and patients receiving ≥4 (analogue for IPOC). The definition of study population by Darling et al. [3] served as a model for the current description. The number of balloon inflations, the average duration of inflations and the delay between first and second inflation during PPCI were obtained from our catheterization laboratory’s procedure database. In this electronic database, each balloon inflation and its location and duration in the coronary artery tree during the procedure is immediately registered, by a cathlab technician using an angioplasty timer, upon indication by the interventional cardiologist. The delay of first re-occlusion after reflow was defined as the time period between first and second balloon inflation. Clinical indications for multiple balloon inflations in the IRA were: long or multiple lesions requiring >1 pre-dilatation, the placement of ≥2 stents or the need for >1 post-dilatation.

### Study endpoints and follow-up

The primary study endpoint was enzymatic IS assessed by peak CK release and was defined as the highest serum concentration in the 72-h time period after the intervention. The upper limit of normal was 180 U/L. The secondary clinical endpoints included all-cause mortality and MACCE during 4-year follow-up. MACCE was defined as a composite of all-cause mortality, non-fatal myocardial infarction, PCI, CABG or stroke. Survival data for all patients were obtained from municipal civil registries. A questionnaire was subsequently sent to all living patients with specific questions on rehospitalization for MACCE. For patients who suffered an adverse event at another centre, medical records or discharge letters from the other institutions were systematically reviewed.

### Statistical analysis

Continuous variables are presented as mean ± standard deviation (SD) or median with interquartile range (IQR), depending on normal distribution and were compared using the Student *t* test or Mann–Whitney *U* test as appropriate. Categorical variables are presented as percentages and were compared using the Chi-square test. Univariable and multivariable linear regression analyses were performed to evaluate the relationship between number of balloon inflations with peak CK after logarithmic transformation. In multivariable analyses the variables age, gender, diabetes, number of diseased vessels, symptom-to-balloon time, Rentrop collateral grade, proximal occlusion of either left anterior descending (LAD) or right coronary artery (RCA) and number of balloon inflations were entered into the model. The differences of median peak CK with 95 % confidence intervals (CI) in subgroups were estimated using the Hodges–Lehmann method for the location shift according to the following prespecified variables: gender, age, diabetes, hypercholesterolemia, hypertension, collaterals, vessel disease, culprit artery, delay first re-occlusion, and symptom-to-balloon time. Patients lost to follow-up were considered at risk until the date of last contact, at which time-point they were censored. Clinical outcomes are presented as Kaplan–Meier survival estimates and were compared using the log-rank test. Multivariable Cox proportional hazard regression analyses were performed to evaluate the relationship between number of balloon inflations and all-cause mortality and MACCE, and are presented as unadjusted and adjusted hazard ratios (HR) with associated 95 % CIs.

The Hodges–Lehmann analysis was performed using SAS version 9.2 (SAS Institute, Cary, NC, USA). All other statistical analyses were performed using SPSS version 20 (SPSS, Inc., Chicago, Illinois, USA). All statistical tests were two-tailed and a *p* < 0.05 was considered to indicate statistical significance.

## Results

### Patient characteristics

**Fig. 1:**
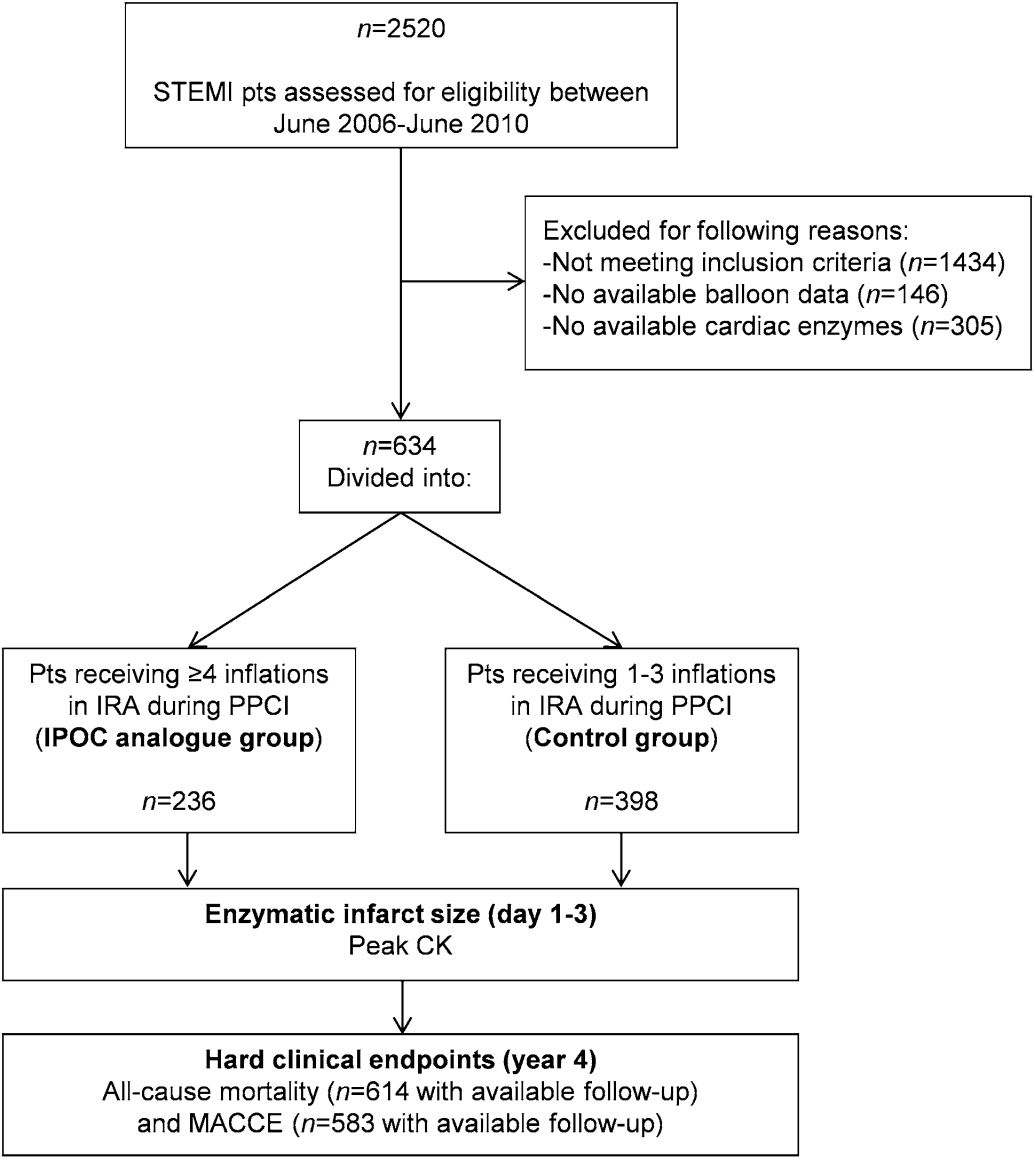
Study flow diagram. *CK* creatine kinase, *IPOC* ischemic postconditioning, *IRA* infarct-related artery, *MACCE* major adverse cardiac and cerebrovascular events, *PPCI* primary percutaneous coronary intervention, *STEMI* ST-segment elevation myocardial infarction

Between June 2006 and June 2010 a total of 2,520 STEMI patients presented to our institution for PPCI, of which 1,086 met the eligibility criteria. Of these, patients without available cardiac enzymes (*n* = 306) and patients without available balloon inflation data (*n* = 146) were excluded. Thus, the current analysis included 634 patients (*n* = 398 in the control group and *n* = 236 in the IPOC analogue group) (Fig. 1). Baseline, angiographic, and procedural characteristics are listed in Table 1. Patients in the IPOC analogue group were older, were less frequently smokers, had less single-vessel and more multivessel disease, and had a tendency of having more Rentrop grade 3 collaterals compared to controls.

**Table 1:** Baseline, angiographic, and procedural characteristics

Variables	Control group (*n* = 398)^a^	IPOC analogue group (*n* = 236)^b^	*p* value
Clinical characteristics and risk factors			
Age (years)	59.9 ± 13.0	62.8 ± 12.6	0.006
Male	75.1	71.6	0.33
Hypertension	38.4	41.0	0.51
Diabetes	10.3	9.7	0.82
Hypercholesterolemia	34.4	36.1	0.67
Current smoker	49.0	39.8	0.03
Family history	35.6	36.3	0.86
Angiographic and procedural characteristics			
Infarct-related artery			
LAD	40.7	36.9	0.34
LCx	17.8	16.1	0.58
RCA	41.5	47.0	0.17
No. of diseased vessels			
1	64.1	44.5	<0.001
2	19.6	30.5	0.002
3	14.6	19.1	0.14
Left main, any^c^	1.8	5.9	0.005
Collaterals			
Rentrop flow grade 0	81.4	77.7	0.27
Rentrop flow grade 1	11.7	10.0	0.52
Rentrop flow grade 2	5.4	8.3	0.15
Rentrop flow grade 3	1.5	3.9	0.06
TIMI flow in culprit artery before PCI			
TIMI 0	88.9	89.4	0.86
TIMI 1	11.1	10.6	0.86
Symptom onset-to-balloon time			
≤3 h	41.3	35.7	0.19
3–6 h	39.7	39.5	0.97
6–12 h	14.4	17.1	0.38
>12 h^e^	4.6	7.6	0.14
Symptom onset-to-balloon time (h)	3.3 (2.4–5.1)	3.8 (2.4–5.9)	0.19
Glycoprotein IIb/IIIa inhibitor use	30.2	36.0	0.13
Adenosine use	0.5	0.8	0.60
Balloon inflation data			
Average balloon inflation time (s)	11.7 (9.5–15.0)	11.6 (9.7–15.7)	0.49
Average balloon inflation pressure (atm)	16.0 (14.0–19.3)	15.0 (12.8–17.0)	<0.001
Delay 1st re-occlusion after reflow (min)	4.0 (2.0–7.0)	3.0 (1.0–6.0)	0.03
Medication during hospitalization in interventional centre^d^			
Aspirin	99.7	100	0.44
Thienopyridine	100	100	–
ACE-i/ARB	3.8	5.0	0.48
β-blocker	50.9	48.6	0.59
Statin	85.1	86.3	0.69
Calcium channel blocker	5.7	7.3	0.44
Diuretic	1.1	1.4	0.76
Nitrate	24.4	25.0	0.87

Data are presented as %, mean (SD) or median (IQR)

*MACCE* major adverse cardiac events, *ACE-i/ARB* angiotensin-converting enzyme inhibitor or angiotensin II receptor blocker, *LAD* left anterior descending coronary artery, *LCx* left circumflex coronary artery, *PCI* percutaneous coronary intervention, *RCA* right coronary artery, *TIMI* thrombolysis in myocardial infarction

^a^Patients receiving 1–3 balloon inflations

^b^Patients receiving ≥4 balloon inflations

^c^Left main only or in combination with 1-, 2- or 3-vessel disease

^d^Percentages upon transfer to peripheral hospital after percutaneous coronary intervention

^e^For patients with ischemic times >12 h, median symptom onset-to-balloon time (with interquartile range) was 16.1 h (13.1–21.5) in the control group (*n* = 17) and 16.5 h (14.1–21.6) in the IPOC analogue group (*n* = 16)

### Balloon inflation data

**Fig. 2:**
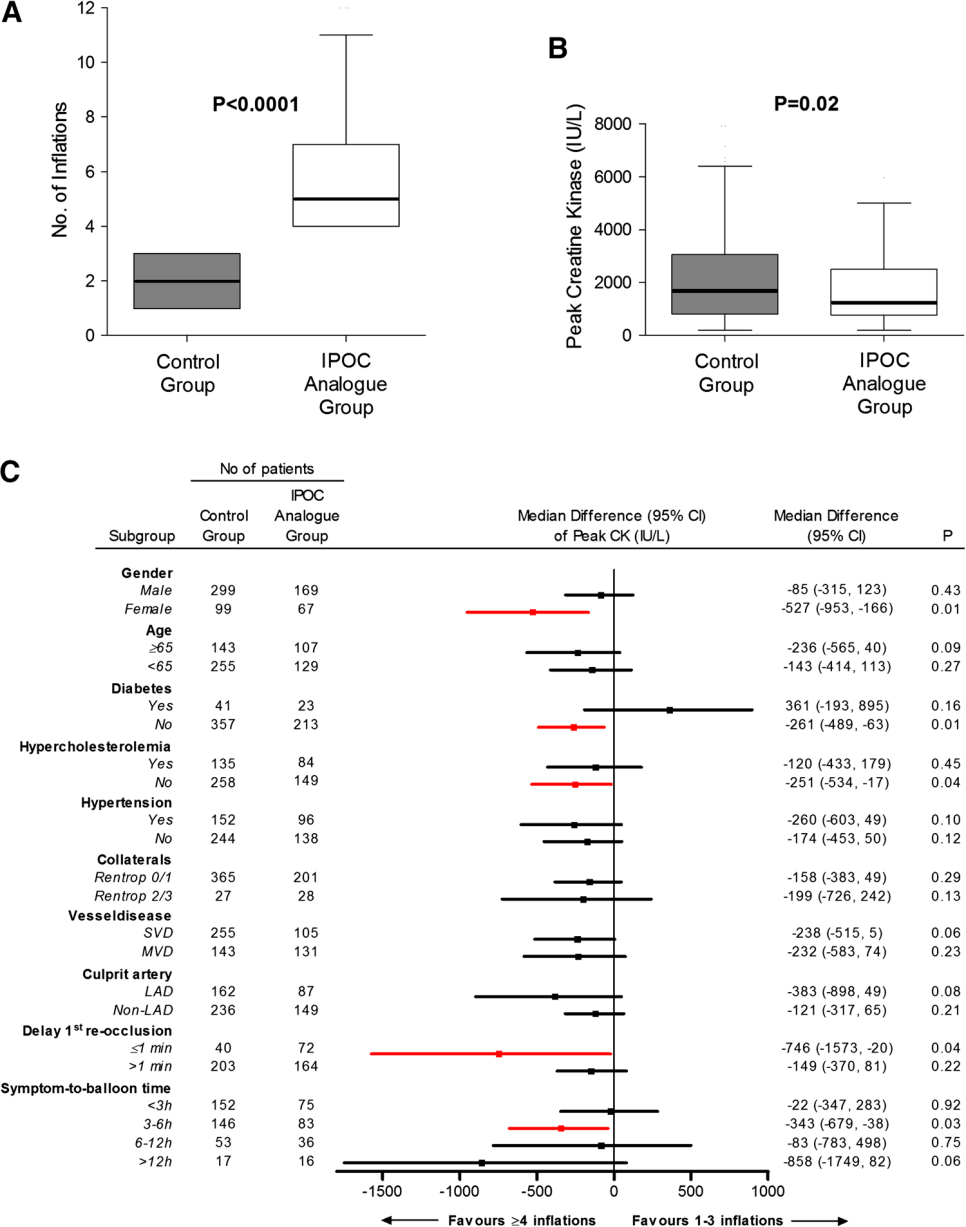
Balloon inflations and enzymatic infarct size. Number of inflations (**a**), peak creatine kinase release in the postconditioning analogue group versus controls in the overall study group (**b**) and differences of median peak enzyme release with 95 % confidence intervals between study groups according to relevant patient subgroups (**c**). The reference for the difference is 1–3 inflations, thus, a negative difference indicates a lower peak creatine kinase in those with ≥4 inflations (**c**). *CI* confidence interval, *CK* creatine kinase, *IPOC* ischemic postconditioning, *LAD* left anterior descending, *MVD* multivessel disease, *SVD* single-vessel disease

The patients in the IPOC analogue group received a median of 5 balloon inflations during PPCI, compared with 2 inflations in controls (*p* < 0.0001) (Fig. 2a). The balloon inflation time and pressure in the IPOC analogue group were 11.6 s (9.7–15.7) and 15.0 atm (12.8–17.0), compared to 11.7 s (9.5–15.0) (*p* = 0.49) and 16.0 atm (14.0–19.3) (*p* < 0.001) in the control group, respectively. The delay of first re-occlusion was 3.0 min (1.0–6.0) in the IPOC analogue group, compared to 4.0 min (2.0–7.0) in controls (*p* = 0.03) (Table 1).

### Infarct size

Peak CK release was significantly lower in the IPOC analogue group compared with controls [1,287 (770–2,498) vs. 1,626 (811–3,057) UI/L; *p* = 0.02] (Fig. 2b). The lower peak CK values in the IPOC analogue group appeared to be more pronounced in: women, patients without diabetes or hypercholesterolemia, patients presenting within 3–6 h of symptom onset, and patients with delay of first re-occlusion ≤1 min (Fig. 2c).

### Long-term outcomes

**Fig. 3:**
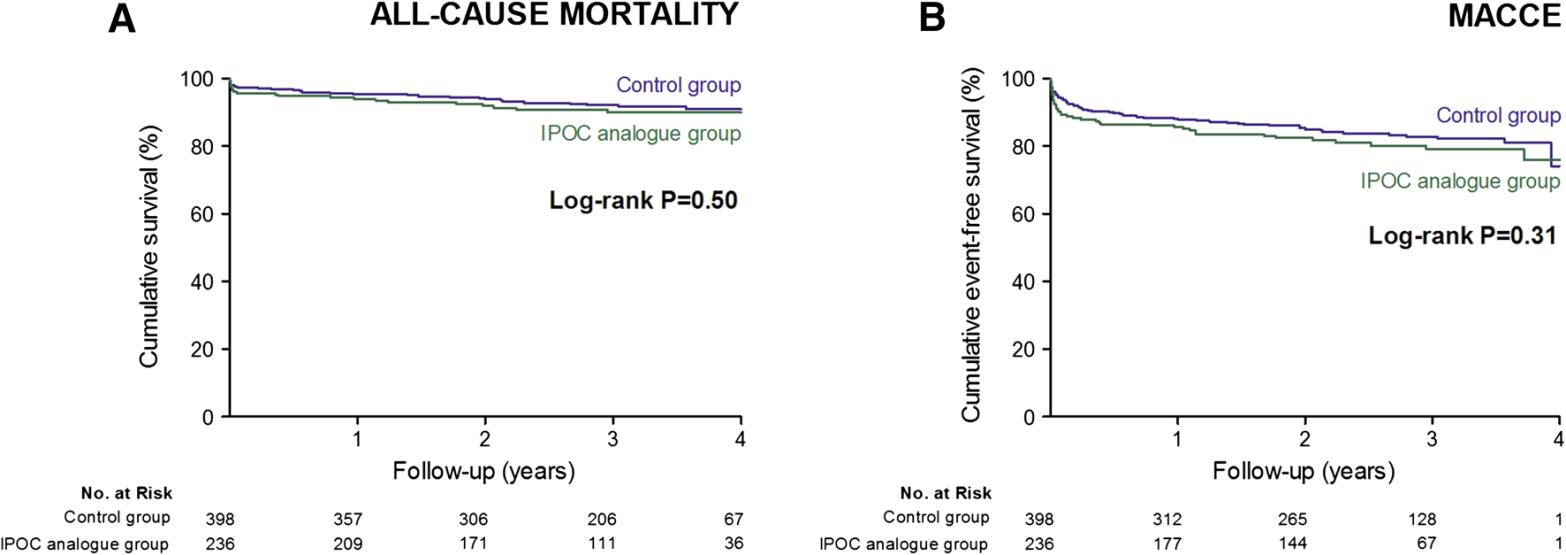
Kaplan–Meier curves of 4-year mortality and major adverse cardiac and cerebrovascular events

Clinical follow-up for mortality was available for 614 patients (97 %). There were 51 deaths during the follow-up: 21 deaths occurred in the IPOC analogue group and 30 deaths occurred in the control group (Kaplan–Meier estimates of 4-year mortality of 10.0 and 9.0 %, respectively; adjusted HR 0.86, 95 % CI 0.44–1.67; *p* = 0.65). Clinical follow-up for MACCE was available for 583 patients (92 %). There were 108 MACCEs during the follow-up: 44 events occurred in the IPOC analogue group and 64 events occurred in the control group (Kaplan–Meier estimates of 4-year MACCE of 24.8 and 26.8 %, respectively; adjusted HR 0.87, 95 % CI 0.57–1.33; *p* = 0.52) (Table 2; Fig. 3).

**Table 2:** Impact of balloon inflations on long-term clinical outcomes

	Unadjusted			Adjusted		
	Hazard ratio	95 % CI	*p*	Hazard ratio^a^	95 % CI	*p*
Mortality	1.21	0.69, 2.11	0.50	0.86	0.44, 1.67	0.65
MACCE	1.22	0.83, 1.79	0.31	0.87	0.57, 1.33	0.52

*CI* confidence interval, *MACCE* major adverse cardiac and cerebrovascular events

^a^The multivariable Cox-regression model included age, gender, diabetes, number of diseased vessels, symptom-to-balloon time, Rentrop collateral grade, proximal occlusion of either left anterior descending (LAD) or right coronary artery (RCA) and number of balloon inflations

## Discussion

The present analysis showed that STEMI patients receiving ≥4 balloon inflations during PPCI displayed lower peak CK values compared with patients receiving 1–3 inflations, corresponding to an enzymatic IS reduction of 21 %. This finding adds further credence to the concept that interrupting the reperfusion procedure with several intermittent episodes of re-occlusions (produced by inflations of the angioplasty balloon) may have acted as a real-world analogue for IPOC and thereby attenuated reperfusion injury. Although the overall beneficial effect remained similar, the lower peak CK values in the IPOC analogue group may be more pronounced in women, patients without diabetes or hypercholesterolemia, patients presenting within 3–6 h of symptom onset and patients with a delay of first re-occlusion ≤1 min. There were no differences in the rates of mortality or MACCE during 4-year follow-up between the IPOC analogue group and the control group in the current study.

### Previous studies

Although it has been demonstrated that IPOC reduced IS with [19, 21, 26] or without [9] an improvement in left ventricular function in the clinical setting, not all studies have been positive [4, 5, 18, 20]. Recently, it was reported that IPOC was not able to reduce IS or improve left ventricular function (assessed by cardiac magnetic resonance imaging) in 79 first-time STEMI patients presenting for PPCI within 12 h of symptom onset [5]. Likewise, Tarantini et al. [20] failed to demonstrate any advantage of IPOC in terms of IS reduction assessed by cardiac magnetic resonance or myocardial reperfusion markers. However, in this trial, the proportion of diabetics was not balanced between study groups (18 % in IPOC group vs. 3 % in controls; *p* = 0.056), which may have blunted the effects of IPOC. Also, it may well be that in patients with symptom-to-balloon times <12 h, a specific IPOC protocol may be ineffective. Importantly, the eligibility criteria differed substantially in the currently available clinical IPOC trials, along with the defined study endpoints [27]. Of these, serum CK release is the most widely used study endpoint [8] and peak CK values strongly correlate with IS and predict cardiac outcomes in STEMI patients treated with PPCI [2, 7]. Currently, no biomarker exists that confirms whether a specific IPOC protocol induced a “postconditioned” state [24]. Nevertheless, assessment of myocardial damage after STEMI is crucial in evaluating the efficacy of reperfusion therapy and predicting prognosis. For instance, most recently Hahn et al. [6] failed to demonstrate any advantage of IPOC in terms of reperfusion markers as assessed by complete ST-segment resolution (>70 %) 30 min post-PCI and postprocedural TIMI flow or myocardial blush grade in a large randomized trial involving 700 STEMI patients. The current results are in line with previous randomized studies, in which IPOC resulted in IS reduction ranging from 27 to 40 % as measured by CK release [19, 21, 23, 26]. The current results are also in agreement with two retrospective studies involving 115 and 85 STEMI patients in which ≥4 [3] or ≥3 [25] balloon inflations during PPCI were associated with lower peak CK values compared with 1–3 or 1–2 inflations. The present study extends these observations by assessing potential confounders and evaluating the association between balloon inflations and long-term clinical outcomes after PPCI in a large cohort of 634 patients. Still, important differences remain with the aforementioned (positive and negative) randomized trials when considering the major determinants of IS. The current study included a proportion of patients with collateral circulation, patients with a wide range of ischemic times, and patients with both LAD and non-LAD infarctions (the latter representing usually smaller areas at risk). In this regard it is noteworthy that the exclusion of patients with Rentrop 2/3 collateral grades (*n* = 55) from the study cohort did not change the beneficial effect of ≥4 balloon inflations during PPCI on enzymatic IS in the IPOC analogue group compared with controls (*p* = 0.04). In addition, we adjusted for these determinants in the regression analysis, including the influence of collateral score.

### Long-term clinical outcomes

The beneficial effect of ≥4 balloon inflations during PPCI on peak CK release in the current study did not translate into improved 4-year clinical outcomes. The reasons for these observations could be related to a number of factors. First, multivessel disease was more often encountered among patients in the IPOC analogue group compared with controls. Consequently, these patients were more likely to receive staged procedures for repeat revascularizations. Second, reperfusion therapy (PCI and thrombolysis) has already reduced mortality after acute myocardial infarction to low levels (4–6 %) [1], making further significant reductions by adjunctive therapies difficult to achieve. In fact, IS may not impact outcomes such as mortality until a threshold IS is achieved. In the current study, IS (peak CK values of 1,287 vs. 1,626 UI/L in study groups) was smaller compared to earlier retrospective studies (1,655 vs. 2,272 UI/L [3]; 2,056 vs. 2,603 UI/L [25]). With the relatively small overall IS and current mortality rates, the present study was likely to be underpowered to discern differences in all-cause mortality. Therefore, no definite conclusions can be drawn regarding the impact of IPOC on long-term outcomes. This is especially true, when considering that most recently both remote ischemic pre- [22] and perconditioning [17] have been found to improve long-term outcomes in prospective randomized trials, including all-cause mortality, in the setting of CABG and STEMI, respectively. Nevertheless, it is reassuring to observe no excess clinical events in the IPOC analogue group in the present study.

### IPOC mechanisms and potential confounders

The current mechanistic paradigm for IPOC invokes the activation of signal transduction pathways by autacoid triggers, which accumulate extracellularly in response to the IPOC stimulus and act on their cell surface receptors or other molecular targets. Ultimately, signaling pathways activated by IPOC probably converge on mitochondria to inhibit the opening of the mitochondrial permeability transition pore, thereby preventing myocardial cell death [12]. Many of these cellular signaling elements might be affected by confounders, co-morbidities or co-medication, as these conditions are associated with fundamental molecular alterations potentially affecting responses to IPOC [12]. Currently, there is insufficient information to judge how and to what extent several confounding factors, including cardiovascular risk factors, gender, and age influence IPOC efficacy in the clinical setting. In this light, we investigated the effect of ≥4 balloon inflations (as a real-world analogue for IPOC) compared with controls in various predefined subgroups. Patients in the IPOC analogue group displayed lower peak CK values compared with controls in nearly all subgroups with a possibly more pronounced effect in women and patients without diabetes or hypercholesterolemia, among others (Fig. 2c). At present, there is no strong evidence whether IPOC-induced protection is gender-dependent [12]. Nevertheless, the current observation is not consistent with a recent meta-analysis in which a pronounced cardioprotective effect of IPOC in male patients was reported [29]. Conversely, although statistically not significant (*p* = 0.16), patients with diabetes displayed higher peak CK concentrations in the IPOC analogue group. This finding is compatible with observations from the pre-clinical setting, in which IPOC-induced cardioprotection was shown to be impaired in diabetes: the IS-limiting effect was lost or required extra cycles of ischemia/reperfusion in various animal models of diabetes [11].

### IPOC protocol and protection

There is consensus that the delay in applying the first re-occlusion after reflow must be relatively short (<1 min) for exerting the beneficial effects of IPOC [24]. In the present study, there was a significant beneficial effect in the IPOC analogue group when this delay was indeed within 1 min, whereas delaying the first re-occlusion above 1 min lowered the efficiency (Fig. 2c). In this regard, one practical issue potentially interfering with IPOC is the use of manual aspiration thrombectomy. This technique usually re-establishes a significant blood flow in the IRA and the delay between applying the procedure and initiating the IPOC protocol might exceed the very short time frame in which IPOC has been shown to be efficient. For this reason, we excluded patients receiving thrombectomy from the present analysis. In the current study, median delay of first re-occlusion after reflow was 3.0 min in the IPOC analogue group. In contrast, in the excluded thrombectomy patients (*n* = 130), median delay was 6.7 min. Indeed, when performing a sensitivity analysis by including the thrombectomy cohort into the current study, median delay of first re-occlusion became 4.0 min in the IPOC analogue group (and even 8.0 min in patients receiving thrombectomy in the IPOC analogue group). Additionally, the beneficial effect of ≥4 balloon inflations during PPCI on enzymatic IS in the IPOC analogue group (*n* = 484) compared to controls (*n* = 280) lost its statistical significance [median peak CK (interquartile range) 1,429 (790–2,761) vs. 1,637 (802–3,114) UI/L; *p* = 0.09] when including the thrombectomy cohort. Future studies need to explore this issue in a randomized setting. On the other hand, despite a median first re-occlusion delay of 3 min (1.0–6.0) in the IPOC analogue group, the use of ≥4 inflations was still associated with lower peak CK values. This observation is in agreement with recent findings in which delaying the IPOC stimulus >1 min after reperfusion onset did not abrogate cardioprotection in the in vivo mouse heart [15] and may suggest that IPOC >1 min may still confer cardioprotection in a clinical setting. Nevertheless, the optimal time window for application of IPOC remains to be determined.

At present, uncertainties remain about the most effective IPOC algorithm in the clinical setting, including number and duration of re-occlusions [24]. In an attempt to define the optimal algorithm in the current cohort, we compared patients receiving a single balloon inflation with the multiple inflation groups. We were not able to ascertain that one particular inflation group was significantly associated with reduced peak CK values (data not shown).

A similar predicament also exists regarding the total duration of the index ischemia. A certain IPOC algorithm applied after an index ischemia of 30 or 45 min significantly reduced IS, but not after an index ischemia of 60 min or longer in a pre-clinical model [16]. Also, with too brief index ischemia, IPOC failed to reduce IS [14]. Hence, there is a need to define the optimal protective protocol for a given duration of the index ischemia. In the current analysis, patients presenting within 3–6 h may have accrued the greatest benefit in the IPOC analogue group. Surprisingly, patients with short ischemic times (<3 h) and those presenting more than 12 h following symptom onset also favored ≥4 balloon inflations. This observation might be especially important as the relationship between extent of myocardial reperfusion injury and very short or very long ischemic times have not been fully elucidated.

### Limitations

The current study suffers from inherent limitations of a non-randomized trial and thus should be considered as a hypothesis-generating post hoc analysis. There were significant differences between study groups in terms of baseline characteristics. To compensate for these differences, we have performed adjustments employing regression and multivariate analyses. PPCI procedures in routine practice were unable to mimic a strict IPOC protocol utilized in randomized trials. Hence, we do not consider ≥4 balloon inflations during PPCI as a surrogate for IPOC. Nonetheless, we limited our analysis to those patients exhibiting a fully occluded IRA and a postprocedural TIMI-3 flow, both representing essential conditions for therapeutic application of IPOC, and were able to demonstrate a beneficial effect on enzymatic IS. Accordingly, our data underscore the need for further prospective studies. We were unable to directly quantify the area at risk in the study population due to the retrospective study design. In an attempt to adjust for (relatively) large volumes at risk, we corrected for proximal LAD or RCA infarctions. The possibility remains that true peak enzyme values were missed. Nevertheless, blood sampling as performed in this study reflects routine clinical practice. Although we did not collect the exact time points of the measurements, we used the highest biomarker concentration in the 72-h time period post-intervention. We were unable to report on co-medication of patients with pre-existing risk factors at the time of presentation, as certain pharmacological agents (e.g., angiotensin-converting enzyme inhibitors) have been shown to impact on IS reduction by IPOC in animal models [12]. However, we reported the prescription rates of pharmacological therapies during hospitalization which was not different between groups, possibly reflecting their chronic use. Moreover, by performing subgroup analyses, we provided insight into the efficacy of multiple balloon inflations in the absence or presence of cardiovascular risk factors. Despite the large number of patients in the current study (*n* = 634) and 97 % available 4-year follow-up, only 51 deaths occurred. This low mortality rate likely reflects the relatively small infarct sizes in our study population. The latter may, in turn be a consequence of not limiting the analysis to patients with proximal lesions (associated with larger volumes at risk). We were unable to report on cardiac death, and the potential misclassification by the use of all-cause mortality in the composite MACCE may have resulted in an underestimation of the relationship between ≥4 balloon inflations during PPCI and outcome. More importantly, an a priori power analysis was not conducted due to the retrospective nature of the study, and with the generally small infarcts and low mortality rates, the present study likely lacks the statistical power to discern differences in all-cause mortality.

## Conclusion

The present study suggests that the use of ≥4 balloon inflations during PPCI reduces enzymatic IS in patients with STEMI under well-defined conditions. This effect may be more pronounced in women, patients without diabetes or hypercholesterolemia, those presenting within 3–6 h, and those with first re-occlusion delay ≤1 min. This hypothesis-generating study suggests that ≥4 inflations during PPCI may have served as a real-world IPOC stimulus and possibly may reflect the phenomenon’s potential in routine clinical practice. Although we present unique data concerning long-term clinical outcomes, adequately powered randomized trials are necessary with strict adherence to IPOC protocol to determine whether a reduction in IS by IPOC translates into improved clinical outcomes.

## Abbreviations

CK: Creatine kinase
IPOC: Ischemic postconditioning
IRA: Infarct-related artery
IS: Infarct size
LAD: Left anterior descending coronary artery
MACCE: Major adverse cardiac and cerebrovascular events
PPCI: Primary percutaneous coronary intervention
STEMI: ST-segment elevation myocardial infarction
